# Micro-fragmented and nanofat adipose tissue derivatives: In vitro qualitative and quantitative analysis

**DOI:** 10.3389/fbioe.2023.911600

**Published:** 2023-01-17

**Authors:** Claudia Cicione, Gianluca Vadalà, Giuseppina Di Giacomo, Veronica Tilotta, Luca Ambrosio, Fabrizio Russo, Biagio Zampogna, Francesca Cannata, Rocco Papalia, Vincenzo Denaro

**Affiliations:** ^1^ Laboratory for Regenerative Orthopaedics, Research Unit of Orthopaedic and Trauma Surgery, Department of Medicine and Surgery, Università Campus Bio-Medico di Roma, Rome, Italy; ^2^ Operative Research Unit of Orthopaedic and Trauma Surgery, Fondazione Policlinico Universitario Campus Bio-Medico, Rome, Italy; ^3^ Operative Research Unit of Endocrinology and Diabetes, Fondazione Policlinico Universitario Campus Bio-Medico, Rome, Italy

**Keywords:** adipose tissue, mesenchymal stromal cells, cell therapy, stromal vascular fraction, regenerative medicine

## Abstract

**Introduction:** Adipose tissue is widely exploited in regenerative medicine thanks to its trophic properties, mainly based on the presence of adipose-derived stromal cells. Numerous devices have been developed to promote its clinical use, leading to the introduction of one-step surgical procedures to obtain minimally manipulated adipose tissue derivatives. However, only a few studies compared their biological properties. This study aimed to characterize micro-fragmented (MAT) and nanofat adipose tissue (NAT) obtained with two different techniques.

**Methods:** MAT, NAT and unprocessed lipoaspirate were collected from surgical specimens. RNA extraction and collagenase isolation of stromal vascular fraction (SVF) were performed. Tissue sections were analysed by histological and immunohistochemical (collagen type I, CD31, CD34 and PCNA) staining to assess tissue morphology and cell content. qPCR was performed to evaluate the expression of stemness-related (*SOX2*, *NANOG* and *OCT3/4*), extracellular matrix (*COL1A1*) and inflammatory genes (*IL1β, IL6* and *iNOS*). Furthermore, multilineage differentiation was assessed following culture in adipogenic and osteogenic media and staining with Oil Red O and Alizarin red. ASC immunophenotype was assessed by flow cytometric analysis of CD90, CD105, CD73 and CD45.

**Results:** Histological and immunohistochemical results showed an increased amount of stroma and a reduction of adipocytes in MAT and NAT, with the latter displaying the highest content of collagen type I, CD31, CD34 and PCNA. From LA to MAT and NAT, an increasing expression of *NANOG*, *SOX2*, *OCT3/4*, *COL1A1* and *IL6* was noted, while no significant differences in terms of *IL1β* and *iNOS* emerged. No statistically significant differences were noted between NAT and SVF in terms of stemness-related genes, while the latter demonstrated a significantly higher expression of stress-related markers. SVF cells derived from all three samples (LA, MAT, and NAT) showed a similar ASC immunoprofile as well as osteogenic and adipogenic differentiation.

**Discussion:** Our results showed that both MAT and NAT techniques allowed the rapid isolation of ASC-rich grafts with a high anabolic and proliferative potential. However, NAT showed the highest levels of extracellular matrix content, replicating cells, and stemness gene expression. These results may provide precious clues for the use of adipose tissue derivatives in the clinical setting.

## 1 Introduction

In the last decade, mesenchymal stromal cells (MSCs) have been extensively investigated in several regenerative medicine applications for a wide variety of diseases (Vadala et al., 2007; Sowa et al., 2011; Pak et al., 2018; Viswanathan et al., 2019; Kabat et al., 2020). MSCs are non-hematopoietic cells of mesodermal origin, with stem-like potential related to self-renewal and multilineage differentiation (Vadala et al., 2008; Kulus et al., 2021). Indeed, MSCs are distributed throughout the body in almost all tissues and are mainly found in the bone marrow (BM), adipose tissue (AT) and umbilical cord (UC). Among them, AT is the most promising source due to its wide distribution, easy retrieval, minimally invasiveness of harvesting and higher number of MSC yield compared to BM (Andia et al., 2019). AT is now considered an endocrine organ capable of releasing several adipokines and is characterized by a complex biology related to the interactions between lipid-rich adipocytes and a heterogeneous population of cells composing the stromal vascular fraction (SVF). Indeed, the SVF includes preadipocytes, fibroblasts, vascular smooth muscle cells, endothelial cells, resident monocytes/macrophages, lymphocytes, and adipose-derived MSCs (ASCs) (Yoshimura et al., 2009; Sharma et al., 2021). ASCs are characterized by a high regenerative potential as reported in pre-clinical studies as well as in several clinical trials on bone and cartilage regeneration and treatment of cardiovascular, gastrointestinal and neurological disorders (Nguyen et al., 2016; Macrin et al., 2017; Bateman et al., 2018; Andia et al., 2019). Similar to MSCs, the repairing/regenerative properties of ASCs have been related to different mechanisms: differentiation towards committed resident cell phenotypes and secretion of immunomodulatory factors, cytokines, growth factors, extracellular vesicles (EVs), as well as other bioactive mediators with anti-apoptotic, antifibrotic and antioxidant effects (Wu et al., 2020; Tilotta et al., 2021; Najar et al., 2022).

The isolation of ASCs requires several steps to disaggregate and digest the tissue. The AT is usually harvested through lipoaspiration from subcutaneous depots (Di Taranto et al., 2015). Subsequent centrifugation leads to density gradient separation of the lipoaspirated material into three layers: an upper oil layer deriving from disrupted adipocytes, an intermediate tissue layer containing the lipoaspirated AT, and a lower liquid/blood fraction. Enzymatic digestion of the intermediate layer permits the isolation of the SVF and thereafter of ASCs through the selection of cells in culture by plastic adherence (Bourin et al., 2013). These procedures (centrifugation, digestion, enzyme inactivation, filtration, etc.), are time-consuming and may increase the risk of contamination due to repeated manipulation of the graft. Therefore, strict regulatory restrictions limit the exploitation of ASCs in the clinical practice.

Interestingly, lipoaspirated AT and its ASC-rich tissue-derivatives (mainly SVF) have promoted the development of several fragmentation techniques able to reduce fat processing time to obtain ASC-rich grafts through single-session surgical procedures. These devices mainly process the AT mechanically, non-enzymatically and enzymatically, and can be performed right at the patient’s bedside (Sharma et al., 2021). Nevertheless, differences in AT processing and resulting grafts lead to different products with diverse characteristics which could affect their application and outcomes in various clinical scenarios.

The aim of this study was to characterize micro-fragmented (MAT) and nanofat AT (NAT) obtained using two different techniques, and to compare these derivatives with raw lipoaspirate (LA) and collagenase-isolated cells (SVF). In particular, differences in terms of expression of stemness and inflammatory genes, surface markers specific for different cell populations and extracellular matrix (ECM) components were investigated.

## 2 Materials and methods

All consumables were purchased from Sigma-Aldrich (Saint Louis, MO, USA) unless otherwise specified. The study was conducted in accordance with the Declaration of Helsinki, and the protocol was approved by the Ethics Committee of Campus Bio-Medico University of Rome (n. 21.19 T). All patients signed a written informed consent.

### 2.1 Patients

LA was obtained from 18 patients (9 males and 9 females; mean age: 63 ± 11 years; no comorbidities) undergoing intra-articular injection of AT-derivatives (mechanically processed) for treating knee osteoarthritis (OA). Briefly, through a 1-cm infraumbilical incision, approximately 200 ml of a tumescent solution (500 ml 0.9% saline, 20 ml mepivacaine 10 mg/ml, 0.7 ml 0.1% epinephrine) were infiltrated in the abdominal subcutaneous fat and liposuction was performed until obtaining approximately 50 ml of tissue. Either a mechanical micro-fragmentation method (Lipogems^©^ Ortho Kit, Lipogems International S.p.A, Milan, Italy) or the modified nanofat method (Persichetti et al., 2017) were used to obtain AT-derivatives from LA.

MAT was obtained using the Lipogems^©^ processing kit as per manufacturer’s instructions (Tremolada et al., 2016b). Briefly, the LA was inserted into the device through a first size reduction filter while allowing a corresponding quantity of saline to flow out toward the waste bag. Subsequently, the device was manually shaken so that stainless steel beads inside emulsified oil residues which were thus removed, together with contaminating blood components and cellular debris, by the gravity counter-flow of saline, while washed fat clusters moved to the top of the device. When the solution appeared clear, the device was turned upside-down by 180°, and a second adipose cluster reduction was obtained by passing the graft through a second-size reduction filter and pushing additional fluid from the lower opening of the device using a 10-ml syringe.

In order to obtain the AT derivatives with the modified nanofat method, the LA was aliquoted in 10 ml Luer-lock syringes and centrifuged at 1,200 g for 3 min. After this step, a 4-phase solution was obtained: the oil in the upper part and the serum in the lower part were removed, carefully keeping the adipose part and the pellet containing the SVF. The resulting tissue was emulsified through energic mix between two 10 ml Luer-lock with a 90° stopcock. 4 ml of the resulting solution were kept for the final product. The remaining material was centrifuged at 1,200 g for 3 min to obtain a solution composed of oil and a lower portion composed of a dense NAT matrix. The final solution consisted in 4 ml previously obtained and 1 ml of NAT.

For both MAT and NAT, 5 ml of final AT derivatives were used for clinical purposes. Waste aliquots obtained by micro-fragmentation (*n* = 6; MAT) or by modified nanofat (*n* = 6; NAT) and aliquots of unprocessed LA (*n* = 6) were used for this study.

### 2.2 Tissue processing

**FIGURE 1:**
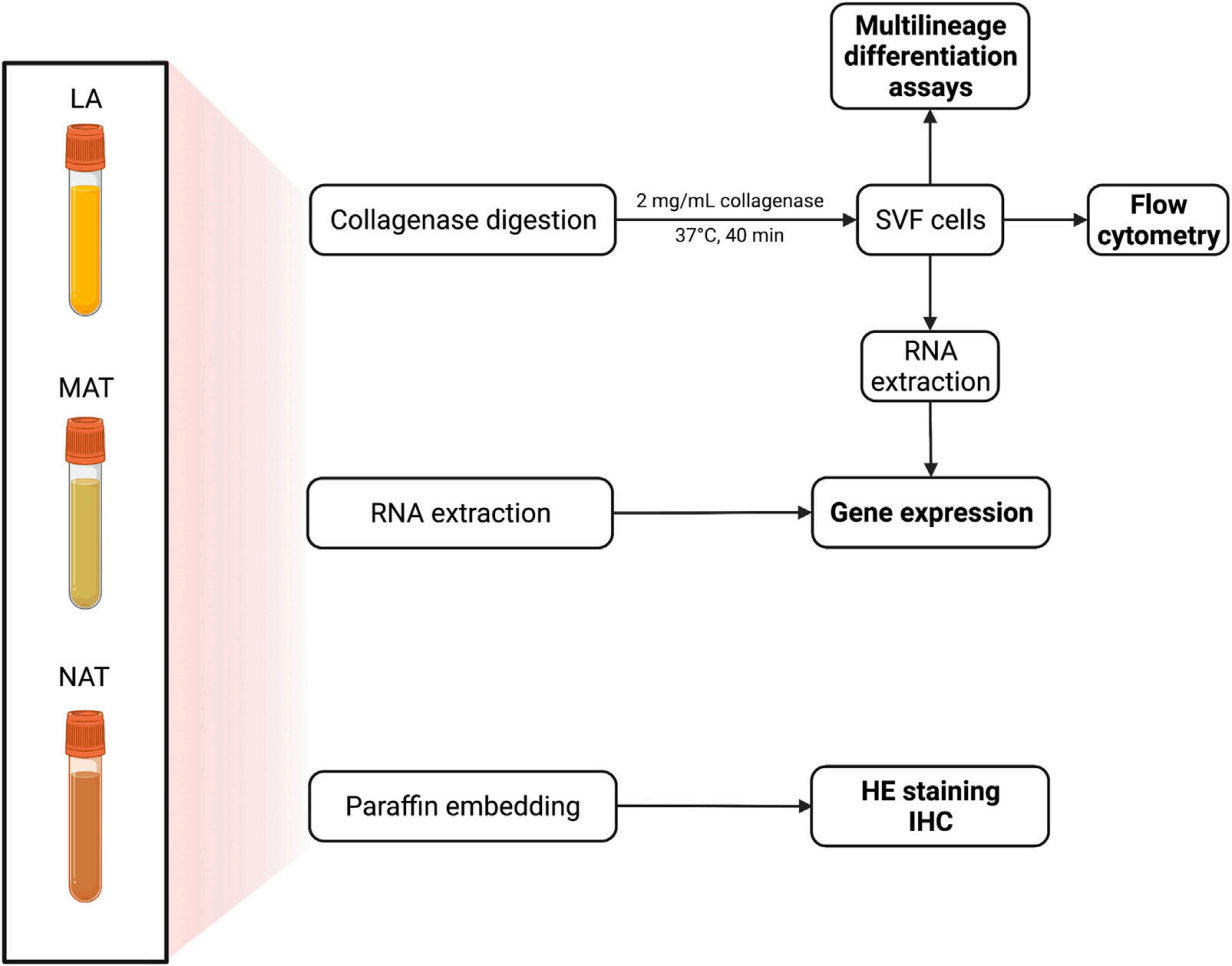
Schematic procedure for the processing of adipose tissue (AT) from lipoaspirate (LA), micro-fragmented (MAT) and nanofat (NAT) samples. Specimens were divided into three aliquots: 3 ml for collagenase digestion; 1 ml for RNA extraction and 1 ml for paraffin embedding. Stromal vascular fraction (SVF) pellet (*n* = 2 for each group) was alternatively used to isolate adipose-derived stem cells (ASCs).

All tissues were processed upon arrival at the laboratory as schematized in Figure 1. Briefly, the samples were equally divided into 3 different aliquots for morphological and immunohistochemical analyses, gene expression evaluation and isolation of SVF, respectively. Considering the different volume, weight and cell density of each AT derivative obtained after processing, the starting LA volume was considered to compare AT by-products among them. For example, if 5 ml MAT were obtained from 25 ml LA and 1 ml of NAT was obtained from 50 ml LA, 5 ml MAT were compared with 0.5 ml NAT.

### 2.3 Immunohistochemical analysis

A part of each specimen was fixed in 10% formalin buffered solution (Bio-Optica, Milan Italy) for 2 h, paraffin-embedded and sectioned into 4 µm-slices. The sections were stained with hematoxylin-eosin (HE; Bio-Optica) following manufacturer’s instructions. Stained slides were analysed with a Nikon A1R + confocal microscope (Nikon Instruments, Tokyo, Japan). Immunohistochemistry (IHC) was performed with the STAT-QTM IHC Staining System for human and animal tissues (INNOVEX, Tokyo, Japan) following manufacturer’s instructions. Negative controls were prepared omitting the primary antibodies. Briefly, after deparaffination 4 µm-sections were incubated for 15 min with a solution of 3% hydrogen peroxide to block endogenous peroxidase activity. Slides were then incubated with primary antibodies (Table 1) for 30 min at room temperature. After washing, sections were incubated with secondary linking antibody for 10 min, washed and incubated with peroxidase (HRP) label for 10 min. After incubation with mixed DAB/substrate solution for 3 min, sections were counterstained with haematoxylin for 1 min, dehydrated and mounted with xylene based mounting media. All tests were performed on at least three sections from each specimen.

**TABLE 1:** Antibodies used for immunohistochemical or cytofluorimetric analysis​.

Product Name	Clone	Catalog Number	Commercial House
Anti-Collagen I antibody	-	AB6308	ABCAM
PCNA Polyclonal Antibody	-	IHC-00012	Bethyl Laboratories
CD31 (PECAM-1)	-	CM 347A	BIOCARE MEDICAL
CD34	-	CM084A	BIOCARE MEDICAL
FITC-CD90	5E10	561969	BD PharmigenTM
FITC-CD45	J33	560976	BD PharmigenTM
FITC-CD105	266	561443	BD PharmigenTM
PE-CD73	AD2	561014	BD PharmigenTM
FITC-Isotype Control	Polyclonal	554001	BD PharmigenTM
PE-Isotype Control	MOPC-21	555749	BD PharmigenTM

### 2.4 Isolation of SVF cells

SVF was isolated from an aliquot of samples from each group (LA, MAT, NAT) through digestion as previously described (Di Taranto et al., 2015). Briefly, fat fraction was separated from each specimen, washed with phosphate-buffered saline (PBS), and then digested with 2 mg/ml collagenase (Worthington) for 40 min at 37 °C under gentle agitation. Collagenase was blocked by adding fetal bovine serum (FBS, Gibco, New York, NY, USA). The digested tissue was filtered through a 70 µm-cell strainer (Becton, Dickinson, and Company, BD, Franklin Lakes, NJ, USA) and the cell suspension was centrifuged at 300 g for 5 min. Distinct aliquots of the resulting SVF were used alternatively for RNA extraction to analyse gene expression levels (Cicione et al., 2016) or for cell culture expansion to characterize isolated ASCs (Bourin et al., 2013).

### 2.5 ASCs culture and characterization

SVF-derived cells were resuspended in alpha-MEM with 15% FBS, 1% penicillin and 1% streptomycin (Gibco) and cultured in humidified atmosphere at 37 °C, with 5% CO_2_. After 24 h, culture medium was replaced to remove non-adherent cells and changed twice a week. Adherent cells were allowed to grow until 80%–90% of confluence and passaged up to the fourth passage. Cell pellets at passage 4 (P4) were used for the experiments.

#### 2.5.1 Flow cytometry analysis

Culture-expanded P4 ASCs were washed and analysed by flow cytometry as previously described (Cicione et al., 2013) using fluorescein isothiocyanate (FITC)- or phycoerythrin (PE)-conjugated antibodies against surface antigen markers specific for MSCs and hematopoietic stem cells (FITC-CD105, FITC-CD45, FITC-CD90, PE-CD73, BD, Table 1). A minimum of 25,000 cell events per assay were acquired using the CytoFlex (Beckman Coulter, Brea, CA, USA) and analyzed using the CytExpert Software (v.2.1, Beckman Coulter).

#### 2.5.2 Multilineage differentiation assays

Adherent cells at P4 were induced toward adipogenic and osteogenic differentiation, as previously described (Cicione et al., 2016). Briefly, adipogenic medium was prepared by adding to DMEM-low glucose 10% FBS, 1 µM dexamethasone, 0.5 mM 3-isobutyl-1-methylxanthine, 10 μg/ml insulin and 100 µM indomethacin. Osteogenic medium composition was prepared by adding to DMEM-low glucose 10% FBS, 0.1 µM dexamethasone, 0.2 mM ascorbic acid 2-phosphate, and 10 mM glycerol 2-phosphate. After 21 days, cell differentiation was assessed morphologically using oil red O staining for cytoplasmic lipid droplets and alizarin red staining for mineralized matrix. Multilineage differentiation was compared with cells cultured in DMEM with 10% FBS. ImageJ software was used to quantify red colour intensity and distribution among selected pictures. Briefly, three random fields were selected from representative sections of each sample and the surface of stained matrix was compared to the whole matrix and expressed as a ratio. Sections were scored by three individuals under blinded conditions.

### 2.6 RNA extraction and gene expression analysis

Total RNA was isolated from 1 ml aliquot of fresh harvested tissues (*n* = 7 for all three AT derivatives) and from collagenase-isolated SVF cells (*n* = 7). Isolation was accomplished using Trizol Reagent (Invitrogen, Waltham, MA, USA), following the manufacturer’s protocol. RNA was quantified at 260 nm using a NanoDrop^™^ spectrophotometer (Thermo Fisher Scientific, Waltham, MA, USA). Total RNA (1 µg) was reverse-transcribed with High-Capacity cDNA Reverse Transcription kit (Applied Biosystems, Waltham, MA, USA). cDNA samples were stored at -20 °C until used. Real-time PCR analysis (qPCR) was performed using TaqMan Gene Expression Assays listed in Table 2 and TaqMan Universal Master Mix II with UNG on a 7900HT Fast Real-Time PCR System (Thermo Fisher Scientific). Expression levels of *NANOG*, *SOX2*, *OCT3/4*, *COL1A1*, *IL1β, IL6* and *iNOS* were investigated. *GAPDH* was used as a housekeeping gene to normalize the amount of target cDNAs of all other genes of interest. For each gene expression, we assigned the value 1 to the lowest level of expression and the other values were measured as relative expression levels (mRNA REL). The expression level of each target gene was calculated using the 2^−ΔCt^ method.

**TABLE 2:** Taqman assays used for gene expression analysis through qPCR.

Product Name	Catalog Number	Commercial House
NANOG	Hs02387400_g1	Thermo Fisher Scientific
SOX2	Hs01053049_s1	Thermo Fisher Scientific
POU5F1 (OCT3/4)	Hs00999632_g1	Thermo Fisher Scientific
IL1β	Hs000174097_m1	Thermo Fisher Scientific
IL6	Hs00174131_m1	Thermo Fisher Scientific
NOS2 (iNOS)	Hs01075529_m1	Thermo Fisher Scientific
GAPDH	Hs03929097_g1	Thermo Fisher Scientific

### 2.7 Statistical analysis

Each experiment was repeated at least three times. All quantitative data are expressed as means ± standard deviations (SD). The statistical analysis of the results was performed using one-way analysis of variance (ANOVA) with Tukey post-test. Results with *p* < 0.05 (*), *p* < 0.01 (**), *p* < 0.001 (***) and *p* < 0.0001 (****) were considered statistically significant. Statistical analysis was conducted using Prism 8 (GraphPad).

## 3 Results

### 3.1 Morphology of AT derivatives

**FIGURE 2:**
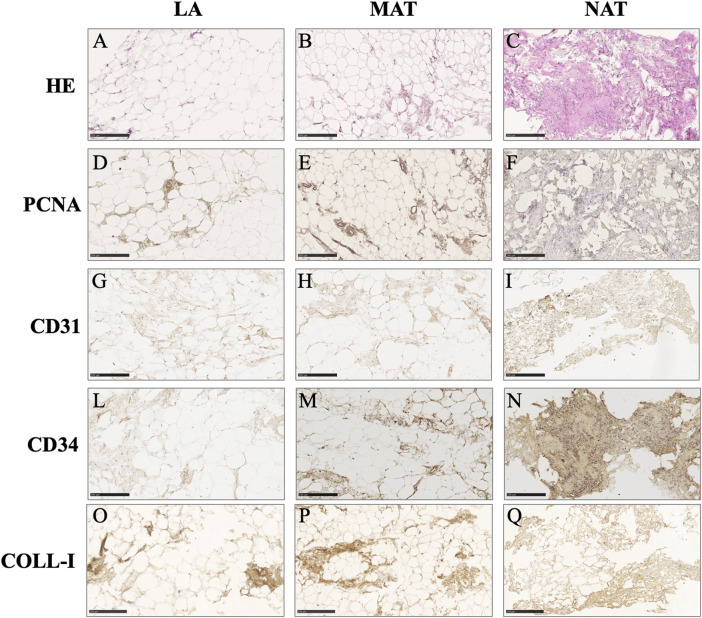
Histochemical and immunohistochemical analysis of lipoaspirate (LA), micro-fragmented (MAT) and nanofat (NAT) formalin-fixed paraffin-embedded sections. Images are representative for each group. **(A–C)**: hematoxylin-eosin (HE); **(D–F)**: Proliferating cell nuclear antigen (PCNA); **(G–I)**: CD31 (PECAM-1); **(L–N)**: CD34; **(O–Q)**: Collagen type I (COLL-I). Scale bars = 250 µm.

Histological analysis of AT derivatives sections showed different tissue architecture and morphology. According to HE staining, only LA samples (Figure 2A) displayed a quite preserved AT morphology with a low degree of adipocyte damage confirmed by intact lobules, spherical cells and intact plasma membranes. On the other hand, MAT samples (Figure 2B) showed a lower number of intact adipocytes compared to LA samples, with increased stroma. Conversely, NAT samples (Figure 2C) almost showed the complete absence of adipocytes and the highest amount of matrix in which an elevated number of cells were dispersed. Immunohistochemistry sections of AT specimens displayed similar expression of proliferating cell nuclear antigen (PCNA), collagen type I (COL1A1), CD31 (PECAM-1) and CD34 in LA (Figures 2D–L, respectively) and MAT (Figures 2E–M, respectively) samples. Interestingly, NAT samples showed the highest amount of PCNA, COL-I, CD31 and CD34 expression (Figures 2F–N, respectively).

### 3.2 Characterization of isolated ASCs

**FIGURE 3:**
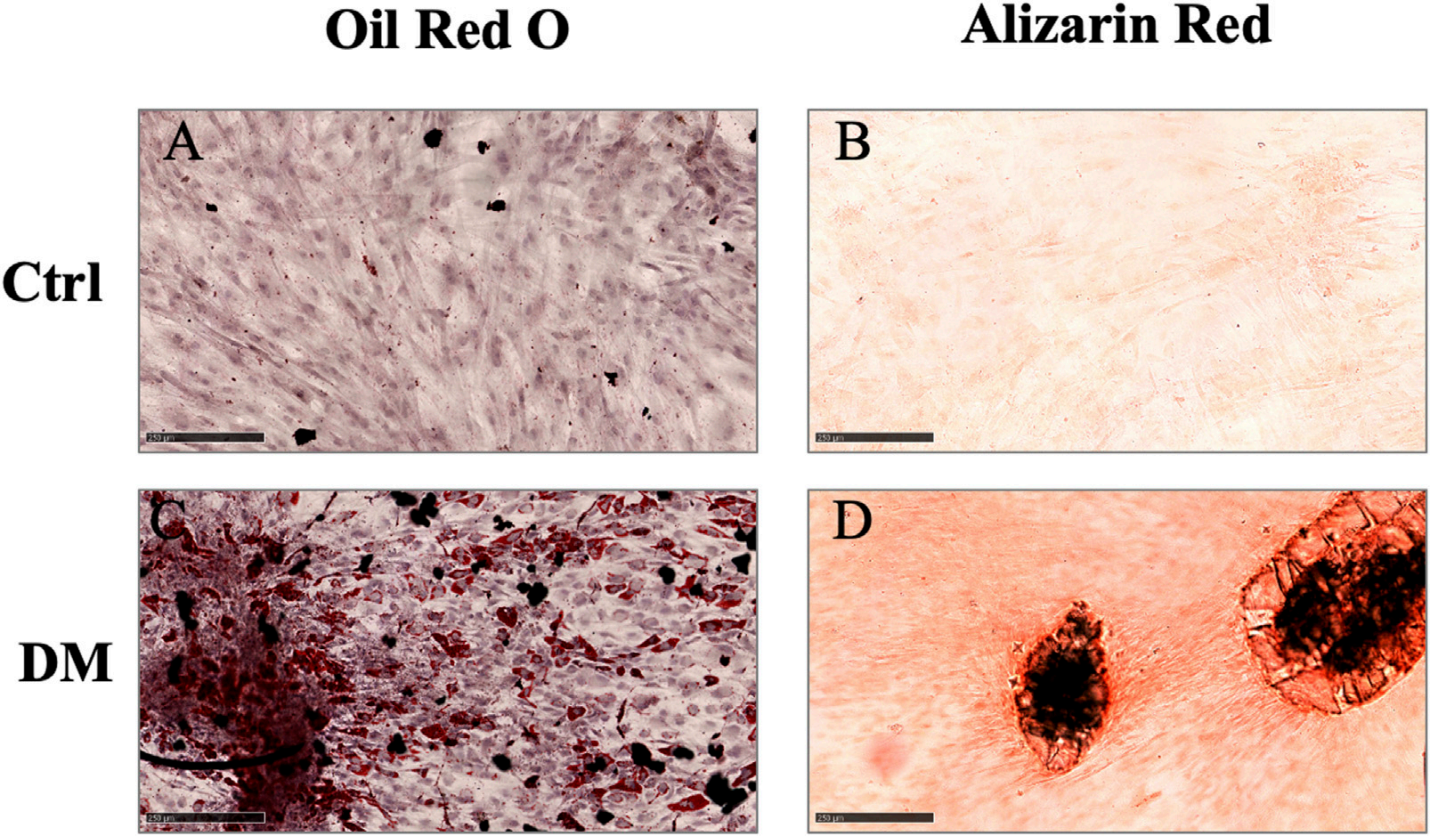
Multilineage differentiation assay. **(A–C)** Oil Red O staining of cells differentiated toward adipocytes (DM), compared to control cells cultured for the same days in DMEM with 10% FBS (Ctrl); **(B–D)** Alizarin Red staining of cells differentiated toward osteoblasts (DM), compared to control cells cultured for the same days in DMEM with 10% FBS (Ctrl). Scale bars = 250 µm.

**FIGURE 4:**
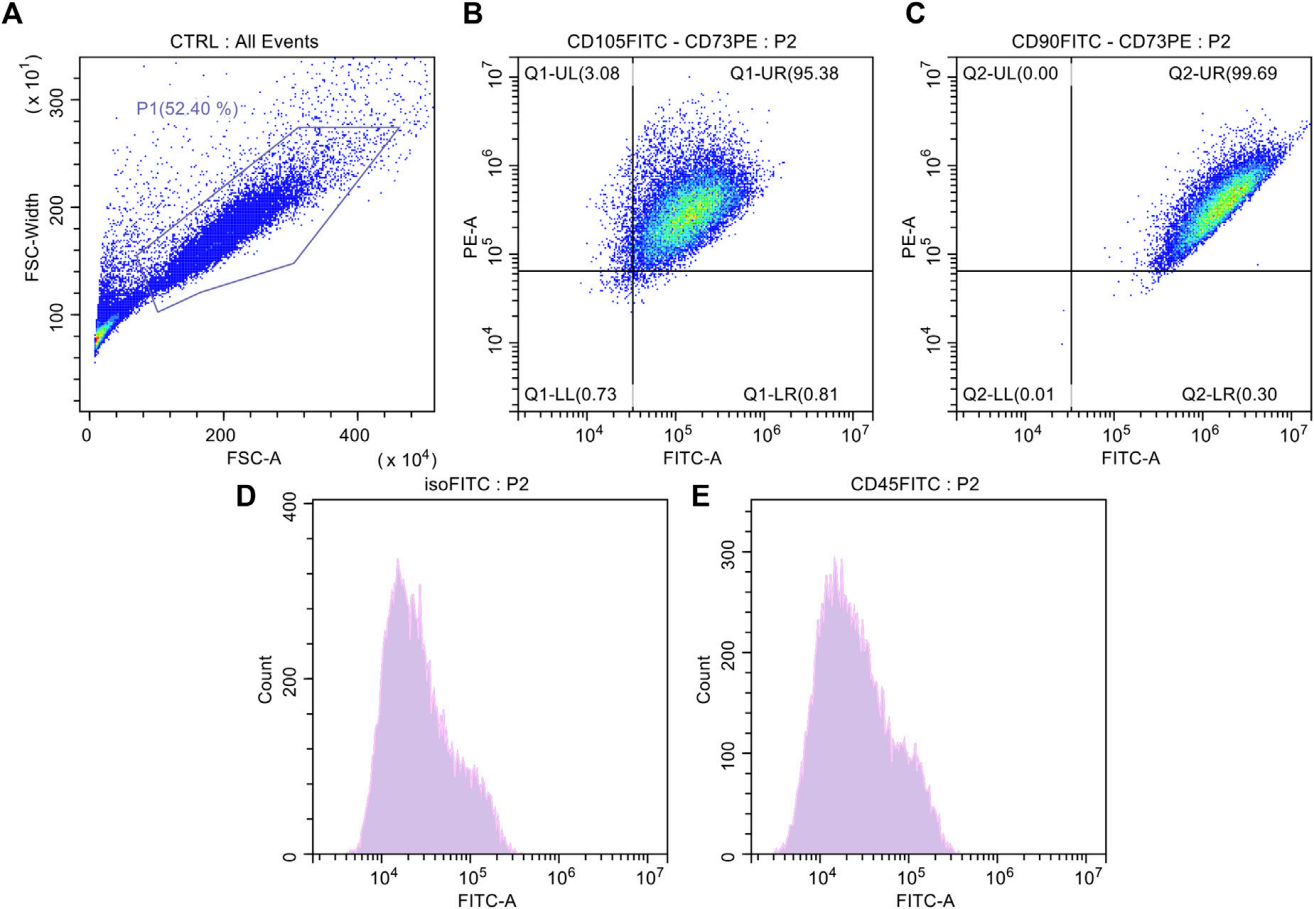
Surface antigen expressions of culture expanded adipose-derived stromal cells (ASCs). **(A)** Control cell population; **(B)** Dot plot of culture expanded ASCs marked with FITC-CD105 and PE-CD73; **(C)** Dot-plot of culture expanded ASCs marked with FITC-CD90 and PE-CD73; **(D)** Histogram of culture expanded ASCs marked with FITC-Isotype Control; **(E)** Histogram of culture expanded ASCs marked with CD45-FITC.

Spindle-shaped bipolar cells were observed in the flasks after collagenase digestion in all AT derivatives under study. The only difference noticed was the starting number of attached cells which was different among MAT, NAT, and LA but without statistical significance (data not shown). After culture expansion, cells were differentiated toward adipogenic and osteogenic lineages and were analysed for the expression of surface markers (CD45, CD73, CD90, and CD105) by flow cytometry. Oil Red O and Alizarin Red staining confirmed that cells derived from the digestion of LA, MAT and NAT were able to differentiate as shown by the red lipid droplets and the red calcium depots without significant differences (Figure 3). Flow cytometric analysis confirmed the immunophenotype of ASCs with expression of CD90, CD105, CD73 cell surface antigens whereas the hematoendothelial marker CD45 was absent (Figure 4).

### 3.3 Differential gene expression profile of LA, MAT, NAT and SVF

**FIGURE 5:**
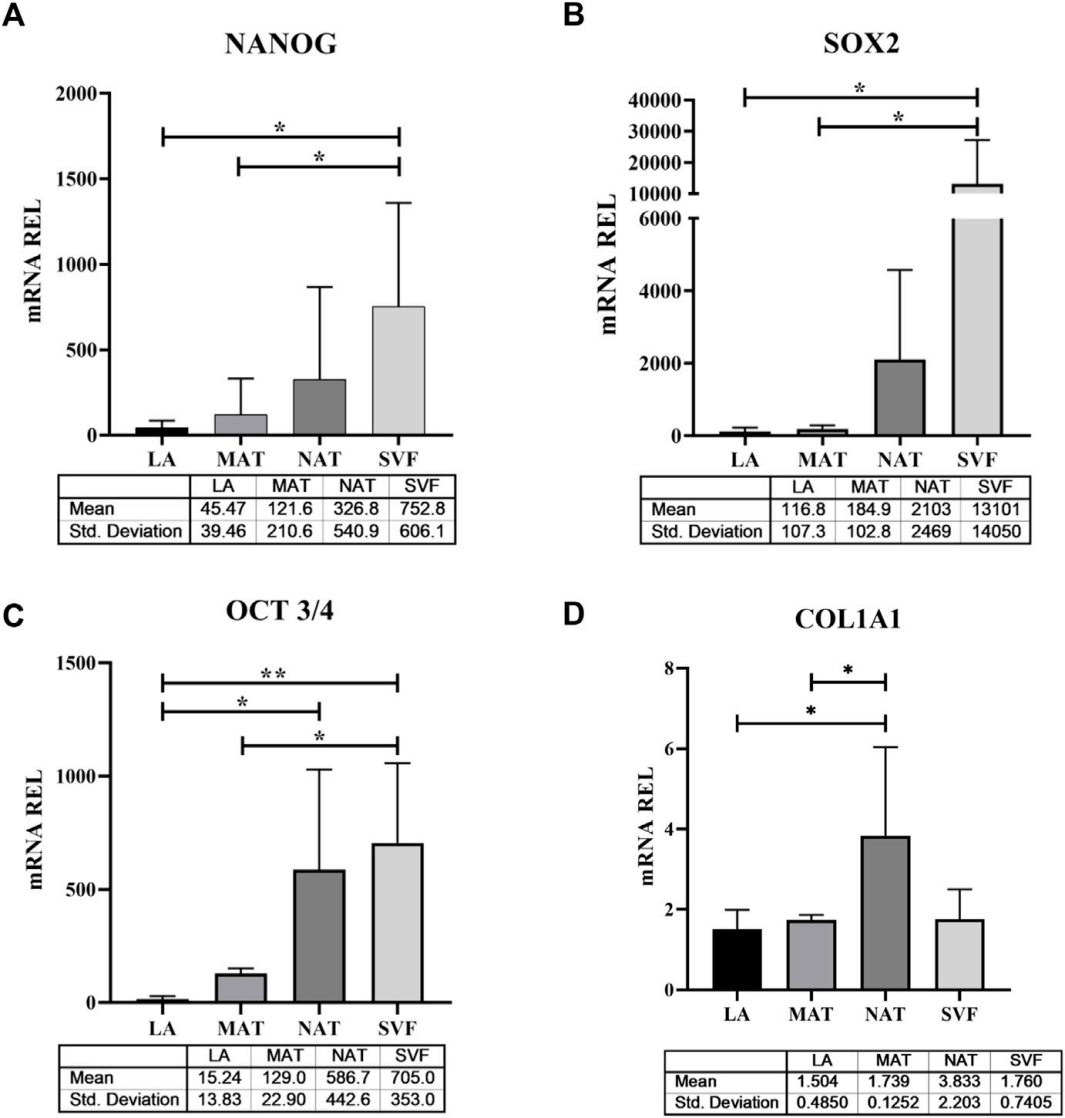
Stemness-related transcription factor and extracellular matrix gene expression in lipoaspirate (LA), micro-fragmented (MAT), nanofat (NAT) and stromal vascular fraction (SVF) samples. **(A)** *NANOG* gene expression levels; **(B)** *SOX2* gene expression levels; **(C)** *OCT3/4* gene expression levels; **(D)** *COL1A1* gene expression levels. Results are expressed as relative quantity (mRNA REL) of gene levels calculated by the 2^−ΔDCt^ method, where the value one was assigned to the lowest level of expression. *p ≤ 0.05; **p ≤ 0.01.

**FIGURE 6:**
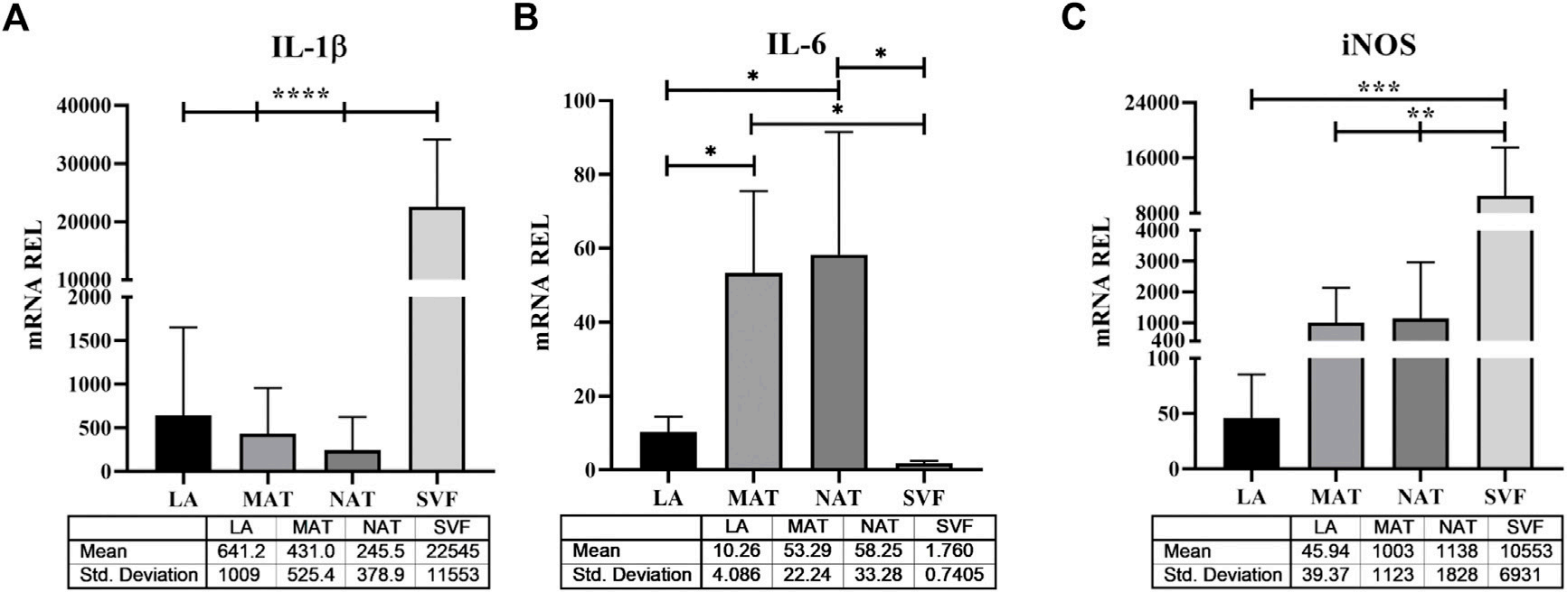
Stress related genes expression in lipoaspirate (LA), micro-fragmented (MAT), nanofat (NAT) and stromal vascular fraction (SVF) samples. **(A)** Interleukin (*IL*)*-1β* gene expression levels; **(B)** IL-6 gene expression levels; **(C)** Inducible nitric oxide synthase (*iNOS*) gene expression levels. Results are expressed as relative quantity (mRNA REL) of gene levels calculated by the 2^−ΔDCt^ method, where the value one was assigned to the lowest level of expression. *p ≤ 0.05; **p ≤ 0.01; ***p ≤ 0.001; ****p ≤ 0.0001.

To explore the molecular features underlying processing differences among the AT derivatives under study, we have comparatively analysed the expression of genes responsible for stemness maintenance (Figure 5), ECM and genes involved in inflammation and oxidative stress (Figure 6). The *NANOG* gene (Figure 5A) was significantly overexpressed in SVF (752.8 ± 606.1) compared to LA (45.47 ± 39.46; *p* = 0.021) and MAT (121.6 ± 210.Six; *p* = 0.044) and without statistical significance compared to NAT (326.8 ± 540.9). Expression levels of *SOX2* (Figure 5B) showed a similar behaviour with an overexpression in SVF samples (13,101 ± 14,050), which was significantly different from LA (116.8 ± 107.3; *p* = 0.048) and MAT (184.9 ± 102.8; *p* = 0.049), but not statistically different from NAT (2103 ± 2469). The stemness essential transcription factor *OCT3/4* (Figure 5C) was overexpressed in SVF (705.0 ± 353.0) followed by NAT (586.7 ± 442.6), both significantly different from LA (15.24 ± 13.83; *p* = 0.007 and *p* = 0.026, respectively). MAT showed an intermediate amount of *OCT3/4* (129.0 ± 22.9) which was significantly different only compared to SVF (*p* = 0.025). In addition, *COL1A1* gene expression (Figure 5D) confirmed IHC findings, showing the highest levels in NAT (3.833 ± 2.203) compared to both MAT (1.739 ± 0.1252, *p* = 0.042) and LA (1.504 ± 0.4850, *p* = 0.014), while not being significantly different from SVF (1.760 ± 0.7405). *IL1β, IL6* and *iNOS* genes were differentially expressed in all AT derivates analysed (Figure 6). In particular, *IL1β* levels (Figure 6A) increased in the following order: NAT (245.5 ± 378.9), MAT (431 ± 525.4), LA (641.2 ± 1,009), and lastly SVF with the highest value (22,545 ± 11,553), significantly different from all the others (*p* ≤ 0.0001). On the other hand, IL6 expression was significantly higher in NAT (58.25 ± 33.28) and MAT samples (53.29 ± 22.24) compared to LA (10.26 ± 4.086; *p* = 0.038 and *p* = 0.019, respectively) and SVF (1.760 ± 0.7405, *p* = 0.023 and *p* = 0.014, respectively). Finally, *iNOS* expression (Figure 6B) was significantly higher in SVF (10,553 ± 6,931) compared with all other samples (LA: 45.94 ± 39.37, *p* = 0.0004; MAT: 1,003 ± 1,123, *p* = 0.002; NAT: 1,138 ± 1828, *p* = 0.001).

## 4 Discussion

For a long time, AT has been mainly regarded as an inactive tissue, mainly serving as an energy storage of the fatty acids contained in mature adipocytes. However, later research demonstrated that AT is indeed an endocrine organ secreting factors involved in several biological and regulatory processes and also contains other cell types including preadipocytes, pericytes, fibroblasts, endothelial cells, hematopoietic cells, macrophages, and ASCs (Gimble et al., 2014). ASCs can be obtained through enzymatic digestion of LA samples, isolated from SVF by plastic adherence and culture expanded *in vitro* (Fraser et al., 2007; Lee et al., 2020; Harrison et al., 2022). Interestingly, ASCs isolated from different subcutaneous fat depots (i.e., abdomen, gluteal region, thigh) show different marker expression and multi-differentiation potential, suggesting that ASCs harvested from different anatomical niches may present unique properties (Ong et al., 2014; Tsuji, 2014; Di Taranto et al., 2015; Vadala et al., 2015). Despite this, ASCs and AT derivatives have already been successfully used in tissue engineering applications and clinical trials thanks to their regenerative potential (Zuk et al., 2001; Si et al., 2019). Indeed, the Food and Drug Administration (FDA) and the European Medicines Agency (EMA) have allowed the use of such AT derivatives for specific therapeutic purposes (Johnson et al., 2017; Raposio and Ciliberti, 2017). In order to be exploited, these AT-derivatives must not be subjected to extensive manipulations, such as collagenase digestion which could cause immune reactions in the host (Kim and Jeong, 2014), thence incompatible with the European Good Manufacturing Guidelines (eGMP) (Regulation (EC) No. 1394/2007 of the Parliament European Union and the European Council). Several devices have been developed to separate and isolate SVF from AT relying on mechanical or physical forces to manipulate the structural integrity of the AT (Oberbauer et al., 2015; Gentile et al., 2019; Rihani, 2019). The final product obtained from these procedures is not a cellular stromal vascular portion, as it occurs from enzymatic digestion, but a combination of cellular debris, blood cells and ECM components (Condé-Green et al., 2016). Among the various non-enzymatic procedures proposed, there are several operator-dependent devices and tools that mechanically dissociate the lipoaspirate (Coccè et al., 2018). Currently, the Lipogems^©^ device is one of the most commonly used in the clinical practice. It consists of a closed device in which intraoperatively lipoaspirated fat is introduced and microfragmented. The final product is a fatty tissue reduced to microfragments (400–600 μm-wide), without oil or blood residues and rich in ASCs (Tremolada et al., 2016a). Tonnard et al. developed a subproduct of AT called “nanofat” by mechanical disintegration (emulsification and filtration), further reducing particle size and making it easily injectable (Gentile et al., 2017; Grünherz et al., 2019). This AT-derivative is devoid of mature adipocytes and contains populations of CD31^+^ and CD34^+^ stromal cells of vascular origin (derived from fragments of arterioles, venules and capillaries), growth factors and naïve cell matrix components (Lo Furno et al., 2017). Preclinical and preliminary clinical studies have demonstrated the feasibility of using nanofat for its reparative capabilities at the graft site by boosting ECM remodeling and angiogenesis, as well as modulating immune system and cell turnover (Tamburino et al., 2016; Chen et al., 2021). However, a few studies have compared the various methods used to obtain AT-derivatives from LA and their effectiveness.

In this study, we analysed the morphology of LA, MAT and NAT through HE staining, which showed that the amount of intact adipocytes drastically decreased from LA, MAT to NAT samples, respectively. Subsequently, IHC analysis showed that NAT samples were characterized by the highest expression of *PCNA*, *COL1A1*, *CD31* and *CD34*, thus demonstrating an increase of cell proliferation, ECM anabolism and vascular marker expression, as well as a higher content of cells and ECM compared to LA and MAT samples. Moreover, we compared the expression of stemness genes *NANOG*, *SOX2* and *OCT3/4* among LA, MAT, NAT and SVF isolated after enzymatic digestion. Interestingly, these markers were significantly overexpressed in SVF compared to LA and MAT, while not being statistically different from NAT. In addition, *OCT3/4* expression level was also significantly upregulated in NAT samples compared to LA. This may suggest that NAT prepared with the modified nanofat technique may produce a graft with a high regenerative potential without the need to perform additional manipulation (i.e., enzymatic digestion). Moreover, NAT also showed the highest gene expression of *COL1A1*, further confirming IHC findings.

Although AT is considered a structural organ, recent evidence has demonstrated its endocrine and paracrine functions exerted by the secretion anti-inflammatory and vasorelaxant adipokines (such as adiponectin, omentin, angiotensin, methyl palmitate and nitric oxide, NO), which contribute to its anti-contractile, anti-inflammatory and antiatherogenic actions (Vezzani et al., 2018; Toussirot, 2020). However, in some circumstances (e.g., obesity, metabolic syndrome) adipokines, cytokines and other factors produced and released by AT may contribute to generate a chronic inflammatory state and promote the development of hypertension, dyslipidaemia, type II diabetes and other metabolic and cardiovascular diseases (Fantuzzi, 2005). Nonetheless, mechanical manipulation of AT itself may activate oxidative stress and inflammation. Our results demonstrated that inflammatory and oxidative stress markers such as *IL1β* and *iNOS* were differentially expressed in the AT derivates under study. We found the lowest levels of *IL1β* and *iNOS* gene expression in NAT and LA, respectively, whereas in both cases SVF showed the highest values compared to all other samples. Therefore, enzymatic digestion performed to obtain SVF resulted in a consistent increase of inflammatory and oxidative stress markers, while NAT processing better maintained their levels compared to MAT. However, our findings also showed that *IL6* was significantly more expressed in NAT and MAT compared to LA and SVF. Differently from IL-1β, IL-6 holds a dual biological role: while it has been traditionally involved in the inflammatory response and release of matrix-degrading enzymes, recent findings have also demonstrated that IL-6 may upregulate anti-catabolic factors, suggesting a protective role (Wiegertjes et al., 2020).

Following enzymatic digestion to obtain SVF, flow cytometric analysis and differentiation towards the adipogenic and osteogenic lineage *in vitro* showed comparable results in all samples derived from the three derivatives under study. This may be explained by the loss of distinct cell features following *in vitro* expansion.

Among the AT derivatives under investigation, NAT showed a dense ECM with a low number of adipocytes and a high content of ASCs and cells of vascular origin, with a reduced expression of pro-inflammatory and oxidative stress-related factors compared to other minimally manipulated and non-enzymatically treated AT-derivatives. Conversely, MAT was mainly composed of mature adipocytes with a lower expression of stemness-related genes, thus resembling the characteristics of LA. Furthermore, the differences in the relative amount of the different types of cells contained in the three set of samples under study may lead to different outcomes.

This study presents some limitations. The main limitation is related to patients’ variability. Indeed, we did not specifically select patients undergoing the regenerative treatment, therefore the collection of AT samples was random. In this regard, we did not evaluate if the presence of comorbidities (diabetes, hypertension, dyslipidaemia) might affect the regenerative potential of AT-derivatives in clinical practice. Furthermore, the majority of LA, MAT and NAT samples analysed did not belong to the same patients (only two LA and MAT samples were matched). However, recent evidence has demonstrated that decreased proliferation and differentiation potential in ASCs has been associated with increasing age, body mass index, diabetes mellitus, exposure to radiotherapy and Tamoxifen, while no significant influence was reported in association with gender, donor site preference, HIV status and chemotherapy. As stated above, our cohort was balanced in terms of male to female ratio (1:1), presented a similar age span, was affected by OA and not by comorbidities (including obesity), did not report a previous history of cancer and related treatments, and underwent fat grafting from the same site through the same incision (Varghese et al., 2017). Therefore, despite patient variability and the small sample size, we expect our data to be effectively representing the biological characteristics of the AT-derivatives under investigation. In addition, some inherent imprecision exists concerning AT derivatives and their nomenclature, mainly due to the wide availability of different harvesting techniques and processing. Indeed, while MAT is defined as such due to the presence of micrometric (∼300 μm) adipose clusters (Tremolada et al., 2016b), no specific component in the nanometric scale is present in NAT. The term NAT was introduced to depict the high degree of emulsification of this by-product and the possibility to inject it through finer sharp needles compared to MAT (Tonnard et al., 2013). Furthermore, the heterogeneity of quality and quantity among analysed AT-derivatives may not accurately represent the biological diversity of cells populating the tissue. Finally, as only a limited number of genes was investigated, analyses of wider inflammatory gene panel would have been needed to better define the immunomodulatory effects of AT derivatives under study.

## 5 Conclusion

Our results indicate that AT-derivatives differ in terms of stemness gene expression, cell content and levels of inflammatory and oxidative stress markers. Interestingly, NAT showed the highest concentration of ASCs and the lowest expression of *IL1β* compared to other grafts. Therefore, considering the cost-effectiveness and ease of processing characterizing the NAT here obtained, such AT-derivative may be ideal for future applications in regenerative medicine. However, further studies are needed to encourage the routine standardized use of NAT in the clinical setting.

## Data Availability

The raw data supporting the conclusions of this article will be made available by the authors, without undue reservation.
